# Structural and biophysical characterization of the Borna disease virus 1 phosphoprotein

**DOI:** 10.1107/S2053230X23000717

**Published:** 2023-02-23

**Authors:** Jack D. Whitehead, Jonathan M. Grimes, Jeremy R. Keown

**Affiliations:** a Division of Structural Biology, Wellcome Trust Centre for Human Genetics, University of Oxford, Oxford, United Kingdom; b Sir William Dunn School of Pathology, University of Oxford, Oxford, United Kingdom; University of Essex, United Kingdom

**Keywords:** bornavirus, phosphoproteins, X-ray crystallography, negative-strand RNA viruses, replication

## Abstract

Bornaviruses are RNA viruses with a mammalian, reptilian, and avian host range. The viruses infect neuronal cells and in rare cases cause a lethal encephalitis. The family *Bornaviridae* are part of the *Mononegavirales* order of viruses, which contain a nonsegmented viral genome. *Mononegavirales* encode a viral phosphoprotein (P) that binds both the viral polymerase (L) and the viral nucleoprotein (N). The P protein acts as a molecular chaperone and is required for the formation of a functional replication/transcription complex. In this study, the structure of the oligomerization domain of the phosphoprotein determined by X-ray crystallography is reported. The structural results are complemented with biophysical characterization using circular dichroism, differential scanning calorimetry and small-angle X-ray scattering. The data reveal the phospho­protein to assemble into a stable tetramer, with the regions outside the oligomerization domain remaining highly flexible. A helix-breaking motif is observed between the α-helices at the midpoint of the oligomerization domain that appears to be conserved across the *Bornaviridae*. These data provide information on an important component of the bornavirus replication complex.

## Abstract 2

The phosphoprotein is an essential component of the replication complex in bornaviruses. Here, using X-ray crystallography, the structure of the phosphoprotein from Borna disease virus 1 is presented and its flexibility in solution is characterized.

## 1. Introduction

Mammalian Borna disease virus 1 (BoDV-1) is a pathogen in the *Bornaviridae* family that causes neurological complications, and in some cases encephalitis and death (Hoffmann *et al.*, 2015 ▸; Tappe *et al.*, 2021 ▸). Other members of the *Bornaviridae* have been found to infect both avian and reptilian species (Kuhn *et al.*, 2015 ▸; Rubbenstroth, 2022 ▸). Early reports on the virus suggested a causal role in many psychiatric disorders; however, this is no longer widely accepted (Rubbenstroth *et al.*, 2019 ▸).

*Bornaviridae* are members of the order *Mononegavirales*, the members of which are nonsegmented negative-sense viruses (nsNSVs) with a single linear RNA genome (Rubbenstroth *et al.*, 2021 ▸). While most members of the order replicate in the cytoplasm of the infected cell, currently only the *Bornaviridae* and a subset of plant-infecting *Rhabdo­viridae* have been described to replicate in the cell nucleus (Jackson *et al.*, 2005 ▸; Rubbenstroth *et al.*, 2021 ▸).

**Figure 1:**
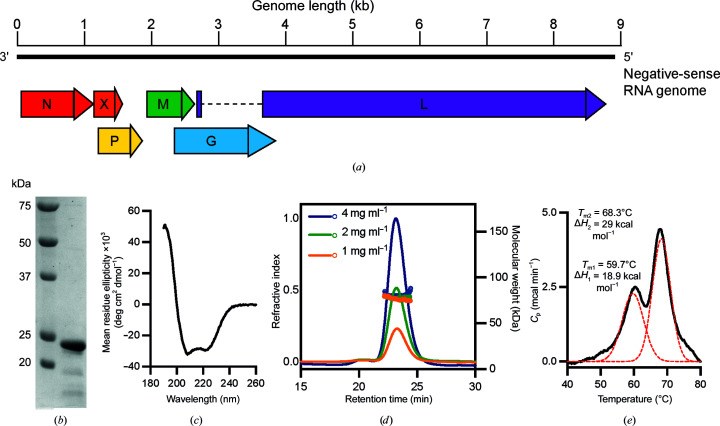
Biophysical analysis of the BoDV-1 phosphoprotein. (*a*) Annotated bornavirus genome and the location of protein-coding transcripts. (*b*) SDS–PAGE analysis of the purified BoDV-1 phosphoprotein. (*c*) Circular-dichroism spectrum of the BoDV-1 phosphoprotein. (*d*) SEC-MALLS analysis of the BoDV-1 phosphoprotein at three protein concentrations. (*e*) Differential scanning calorimetry of the BoDV-1 phosphoprotein with two-state fitted curves to determine the melting temperature and heat capacity of each transition.

BoDV-1 has the smallest genome of the *Mononegavirales* order, at 8.9 kilobases, which encodes at least six viral proteins (Fig. 1 ▸ *a*; Rubbenstroth *et al.*, 2021 ▸). Similar to other nsNSVs, the virus encodes proteins which facilitate cellular entry (glycoprotein), virion formation (matrix protein) and genome transcription and replication (nucleoprotein, large protein and phosphoprotein). BoDV-1 contains a viral accessory protein (X protein) which is poorly understood and is implicated to have a range of functions (Wensman *et al.*, 2013 ▸; Fujino *et al.*, 2012 ▸). Together, the nucleoprotein (N), large protein (L), phosphoprotein (P) and the viral RNA form a viral ribo­nucleoprotein (vRNP) complex. The viral N protein encapsulates the viral genome, protecting it from cellular nucleases. The L protein is an RNA-dependent RNA polymerase (RdRp) and carries out transcription and genome replication. The P protein is a cofactor of the L protein and appears to also interact with the viral X protein (Fujino *et al.*, 2012 ▸).

In members of the *Mononegavirales* order, the P protein plays two discrete roles while in complex with the L protein. One function is to tether the L protein to the RNP complex via interaction with RNA-bound N (Green & Luo, 2009 ▸). The second function is to bind and recruit RNA-free N (also denoted N^0^), which is added to newly synthesized RNA (Leyrat *et al.*, 2011 ▸; Yabukarski *et al.*, 2014 ▸; Guryanov *et al.*, 2016 ▸; Leung *et al.*, 2015 ▸; Renner *et al.*, 2016 ▸; Zhu *et al.*, 2017 ▸; Aggarwal *et al.*, 2018 ▸; Dong *et al.*, 2022 ▸). Phosphoproteins appear to have a globally conserved structure with a central oligomerization domain flanked by largely unstructured N- and C-termini (Cardone *et al.*, 2021 ▸).

Structures of phosphoprotein oligomerization domains of viruses within the *Mononegavirales* order have been determined both alone and in complex with other viral proteins. The most widely studied are those from the *Paramyxoviridae*, which have been shown to form tetramers with oligomerization mediated by a large coiled coil (Blocquel *et al.*, 2014 ▸; Bloyet *et al.*, 2019 ▸; Bruhn *et al.*, 2014 ▸; Communie *et al.*, 2013 ▸; Cox *et al.*, 2013 ▸; Tarbouriech *et al.*, 2000 ▸). This tetrameric assembly has also been observed in complex with the viral L protein (Abdella *et al.*, 2020 ▸; Xie *et al.*, 2021 ▸). The oligomerization domain of *Filoviridae* forms trimers under certain crystallization conditions (Bruhn *et al.*, 2017 ▸; Zinzula *et al.*, 2019 ▸), while forming tetramers when in complex with the L protein (Yuan *et al.*, 2022 ▸). There are two examples of *Rhabdoviridae* P-protein oligomerization domains from rabies virus and vesicular stomatitis virus, which each form different dimeric assemblies (Ding *et al.*, 2006 ▸; Ivanov *et al.*, 2010 ▸). One *Pneumoviridae* oligomerization domain has been determined in isolation (Leyrat *et al.*, 2013 ▸) and in complex with the L protein (Cao *et al.*, 2020 ▸; Pan *et al.*, 2020 ▸), with all of the structures showing oligomerization as a short, coiled-coil tetramer.

In the present study, we present the structure of the phosphoprotein oligomerization domain from BoDV-1, revealing a tetrameric assembly in solution and in a crystal structure. Analysis of the structure shows a helix-breaking motif, which combined with the pitch of the helix causes displacement of the helices. Small-angle X-ray scattering experiments and *in silico* model generation suggest the unresolved N- and C-termini to be highly flexible in solution. Our study shows a close similarity to phosphoproteins from paramyxoviruses.

## 2. Methods

### 2.1. Protein cloning and purification

The mammalian BoDV-1 phosphoprotein (UniProt P0C799) with an N-terminal His_8_ tag and TEV protease site was expressed in Sf9 cells from a codon-optimized gene cloned into the MultiBac system. Cells were grown in Sf-900 II serum-free medium (Gibco). 72 h after infection, the cells were harvested and resuspended in wash buffer [50 m*M* HEPES pH 7.5, 500 m*M* NaCl, 0.05%(*w*/*v*) *n*-octyl β-d-thioglucopyranoside, 10%(*v*/*v*) glycerol, 1 m*M* dithiothreitol, 20 m*M* imidazole] supplemented with one protease-inhibitor tablet (Sigma) and 5 mg RNAse. The cells were then lysed by sonication prior to clarification by centrifugation. The supernatant was applied onto Ni–NTA and washed with wash buffer before the protein was eluted with wash buffer supplemented with 500 m*M* imidazole. The desired fractions were then pooled and 0.3 mg TEV protease was added. The sample was dialysed overnight against 20 m*M* HEPES pH 7.5, 300 m*M* NaCl, 1 m*M* dithiothreitol, 5%(*v*/*v*) glycerol.

The cleaved protein was then diluted to decrease the NaCl concentration to 150 m*M* before being applied onto a 5 ml Q-HP column pre-equilibrated with 20 m*M* HEPES pH 7.5 and eluted with a linear gradient to 20 m*M* HEPES pH 7.5, 500 m*M* NaCl. Fractions containing the purified protein were pooled, concentrated and applied onto a Superdex 200 Increase 10/300 column equilibrated with 20 m*M* HEPES pH 7.5, 150 m*M* NaCl, 5%(*v*/*v*) glycerol. Samples could not be frozen without aggregation.

### 2.2. Circular dichroism

BoDV-1 phosphoprotein was extensively dialysed against a buffer consisting of 10 m*M* sodium phosphate pH 7.4 prior to data collection. Spectra were collected from protein at a concentration of 0.05 mg ml^−1^ in a quartz cuvette with a 0.1 cm path length using a Jasco J-815 spectrophotometer. Data were corrected with a buffer spectrum scan and the units were converted to mean residue ellipticity.

### 2.3. Differential scanning calorimetry

The protein was dialysed into a buffer consisting of 20 m*M* HEPES, 150 m*M* NaCl. 400 µl protein at a concentration of 1.2 mg ml^−1^ (53 µ*M*) was applied onto a Malvern VP Capillary DSC. Protein and running buffer controls were heated at a rate of 1°C min^−1^. The data were processed in *OriginPro* (Origin­Lab) using a non-two-state model to determine the thermal transition midpoint (*T*_m_) and heat capacity (*C*_p_).

### 2.4. Size-exclusion chromatography coupled to multi-angle laser light scattering

Size-exclusion chromatography coupled to multi-angle laser light scattering (SEC-MALLS) analysis was performed using a Shimadzu chromatography system connected to a HELEOS-II eight-angle laser light-scattering detector and an Optilab T-rEX refractive-index detector (Wyatt Technologies). All analysis was carried out at 20°C. 100 µl of each sample was applied onto a Superdex 200 10/300 column pre-equilibrated in a buffer consisting of 20 m*M* HEPES pH 7.5, 150 m*M* NaCl, 5%(*v*/*v*) glycerol. Data were processed using the *ASTRA* suite of software (Wyatt Technologies).

### 2.5. Crystallization

100 nl of freshly purified full-length BoDV-1 phospho­protein at a concentration of 6 mg ml^−1^ was mixed in a 1:1, 2:1 or 1:2 ratio with a set of sitting-drop crystallization screens and placed at room temperature. Crystals with a plate morphology appeared after approximately 24 days from a condition consisting of 0.2 *M* ammonium tartrate, 20%(*w*/*v*) PEG 3350. Crystals were harvested into a buffer consisting of reservoir solution supplemented with 25%(*v*/*v*) glycerol as a cryoprotectant and were cooled in liquid nitrogen. Crystallization conditions are summarized in Table 1 ▸.

### 2.6. Structure determination and refinement

All diffraction experiments were carried out on the I03 beamline at Diamond Light Source. Data-collection and processing statistics are presented in Table 2 ▸. The data were initially reduced with *autoPROC* and *STARANISO* (Von­rhein *et al.*, 2011 ▸). A primary-sequence search of the Protein Data Bank (PDB) yielded no models with sufficient similarity for use as a search template for molecular replacement. Attempts were made to use *ab initio* models from *ARCIMBOLDO* (Caballero *et al.*, 2018 ▸); however, these were unsuccessful. To leverage developments in protein prediction methods, a model was generated using the *ColabFold* implementation of the *AlphaFold*2 algorithm (Mirdita *et al.*, 2022 ▸).

Using *Phaser* (McCoy *et al.*, 2007 ▸), initial solutions were found with a single chain in space group *P*42_1_2. These solutions showed the features that would be expected in a correctly phased map, including connected helical density and molecules which would pack to form a lattice; however, subsequent refinement of these models did not decrease the *R*_free_ or improve the map quality. The data set was expanded to *P*1 and molecular replacement was run again, searching for a truncated tetrameric oligomerization domain comprising residues 95–145. This solution contains two antiparallel tetramers. The *P*1 solution was then refined with iterative rounds of manual model building in *Coot* (Casañal *et al.*, 2020 ▸) and refinement in *phenix.refine* (Afonine *et al.*, 2018 ▸). Refinement in *Phenix* was carried out with torsion-angle non­crystallographic symmetry restraints and two TLS groups describing the region N- and C-terminal to the helix-breaking motif for each chain.

**Figure 2:**
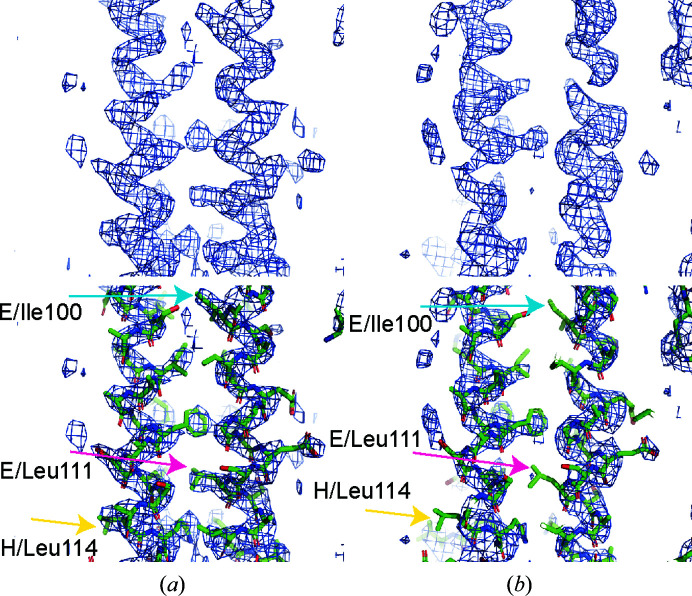
Electron-density map quality. 2*F*_o_ − *F*_c_ electron density is shown at 1.5σ for maps generated either without twinned refinement (*a*) or with twin refinement (*b*). Maps alone (top) or with the fitted model (bottom) are shown separately for clarity. Residues from chains *E* and *H* are highlighted to illustrate differences in map quality.

Attempts to extend the resolution of the usable data did not lead to a more interpretable map, to an improved fit to the data or to improved model statistics. After the model was complete in space group *P*1, *Zanuda* (Lebedev & Isupov, 2014 ▸) was used to determine whether a higher order space group could be used. No alternate space groups were found that yielded solutions that could be refined satisfactorily. Analysis of space group *P*1 using *phenix.xtriage* suggested that pseudo-merohedral twinning may be present in the data. Refinement using either automatic twin refinement in *REFMAC*5 (Murshudov *et al.*, 2011 ▸) or manual input of the twin law in *phenix.refine* resulted in a decrease in both *R*_work_ and *R*_free_ by 6% using *REFMAC*5 and by 2% using *phenix.refine*. However, the resulting 2*F*_o_ − *F*_c_ electron-density maps were of notably poorer quality compared with the *P*1 maps (Figs. 2 ▸ *a* and 2 ▸ *b*). From these analyses, we concluded that twin refinement should not be included in the final processing of our data.

Final structure-solution and refinement statistics can be found in Table 3 ▸. The data and model have been deposited in the PDB with accession code 8bs7.

### 2.7. Small-angle X-ray scattering

All data were collected on the B21 beamline at Diamond Light Source. Prior to experiments, the protein was buffer-exchanged into a buffer consisting of 20 m*M* HEPES pH 7.5, 150 m*M* NaCl, 5%(*v*/*v*) glycerol. All samples were collected by size exclusion prior to small-angle X-ray scattering (SAXS), in which 50 µl of sample was applied onto a Shodex KW-403 column with a flow rate of 0.16 ml min^−1^. Scattering data were collected at a wavelength of 0.9524 Å and a sample-to-detector distance of 3.7 m, and the data were recorded on an EIGER X 4M detector (Dectris). The data were processed using the *ATSAS*3 software suite (Manalastas-Cantos *et al.*, 2021 ▸).

## 3. Results and discussion

### 3.1. Oligomeric state of the BoDV-1 phosphoprotein

A synthetic gene encoding the full-length BoDV-1 phosphoprotein was cloned and expressed and the protein was purified to a high degree of purity, although it appears to be slightly degraded (Fig. 1 ▸ *b*). To first confirm that the protein was correctly folded and achieve an initial understanding of the fold, we performed circular-dichroism spectroscopy. The spectrum showed minima at 208 and 222 nm and a maximum at 193 nm, which is a characteristic spectrum of a highly α-helical protein (Fig. 1 ▸ *c*). Further analysis of the spectrum using *BeStSel* (Micsonai *et al.*, 2018 ▸) predicts the protein to be 62% helical (good helix-1, 47%; distorted helix-2, 15%) and the rest of the structure to be 6% sheet, 5% turn and 27% other. This initial assessment suggests that the protein may be structured similarly to the *Paramyxoviridae* or *Pneumoviridae* proteins.

To determine the oligomeric state of the protein, we used SEC-MALLS and applied protein at concentrations of 1–4 mg ml^−1^. At all concentrations, we observed a symmetric elution peak with a consistent retention of 23.5 min (Fig. 1 ▸ *d*). The coincident peaks suggest no concentration dependence of the oligomerization. The calculated protein molecular mass of the peak was approximately 82 kDa, which is in good agreement with the predicted molecular weight of a phosphoprotein tetramer of 90 kDa. The mismatch between the calculated and predicted molecular weight may arise from minor degradation of the phosphoprotein, which was observed even in freshly purified sample (Fig. 1 ▸ *b*).

To determine the stability of the protein, we carried out thermal melt experiments using differential scanning calorimetry (DSC). The melt experiment showed two distinct transitions at 59.7 and 68.3°C corresponding to two unfolding events (Fig. 1 ▸ *e*). By analogy to other phosphoproteins, this could correspond to dissociation of the oligomer into protomers and subsequent unfolding of the coiled-coil domain (Bruhn *et al.*, 2017 ▸). Next, we sought to understand the molecular details of the oligomerization of the phospho­protein.

### 3.2. Crystallization of the oligomerization domain

**Figure 3:**
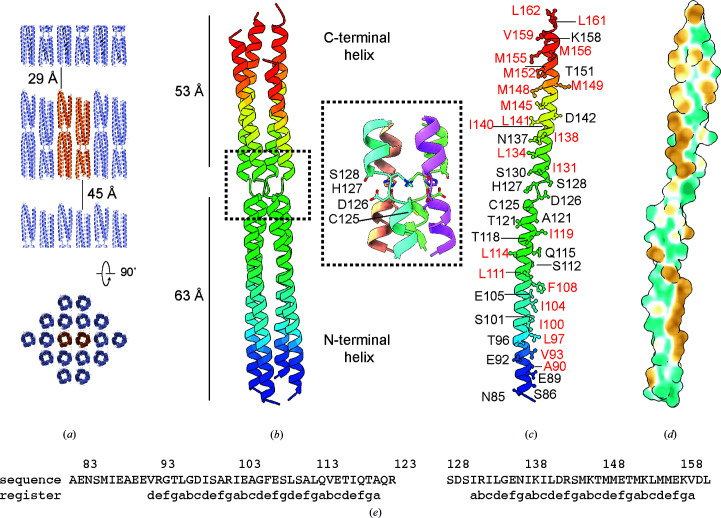
Crystal structure of the BoDV-1 phosphoprotein. (*a*) Packing of the crystalline lattice showing one asymmetric unit (orange) and neighbouring protein tetramers (blue). (*b*) Arrangement of the chains in the tetramer coloured from the N-terminus (blue) to the C-terminus (red). The lengths of the two helices and the positions of the helix-breaking motif are annotated. The inset shows the residues involved in the motif, with the four chains coloured uniquely. (*c*) Residues that stabilize the tetramer by either hydrophobic (red) or electrostatic (black) interactions are annotated. (*d*) Profile of hydrophobicity across the surface of the protein in the same orientation as in (*c*). The surface is coloured from hydrophilic (green) to hydrophobic (gold). (*e*) Repetition of the heptad repeat (a–g) is shown together with the sequence of the oligomerization domain.

We determined the structure of the BoDV-1 phospho­protein using X-ray crystallography. The crystallographic unit cell contains two copies of a P-protein tetramer that are stacked side by side in an antiparallel arrangement (Fig. 3 ▸ *a*). The crystal form contains large gaps between the ordered proteins of 29–45 Å; presumably, this volume partially or fully contains the unresolved N- and/or C-terminal residues. The *A*–*D* tetramer is essentially identical to the *E*–*H* tetramer (r.m.s.d. of 0.31 Å for equivalent C^α^ atoms). Unless stated otherwise, we will discuss the tetramer formed by chains *A*–*D* as this is the most complete tetramer.

Within the *A*–*D* tetramer, residues 85–152, 83–158, 82–162 and 83–162 are ordered in chains *A*, *B*, *C* and *D*, respectively (Fig. 3 ▸ *b*). In good agreement with the circular-dichroism experiment, the ordered regions of the phosphoprotein are almost completely helical in nature. The oligomerization domain is bipartite, with residues 82–124 forming an N-terminal helix of 63 Å in length and residues 129–162 forming a C-terminal helix of 53 Å in length.

The tetramer is maintained through a large buried hydrophobic interface, with each chain of the tetramer burying on average 2764 Å^2^ or approximately 40% of the total surface area of each chain with the three adjacent polypeptides (Figs. 3 ▸ *c* and 3 ▸ *d*). Analysis of the structure shows a classical repeating heptad repeat, with hydrophobic residues forming ‘knobs-in-holes’ packing with hydrophobic residues from adjacent chains (Fig. 3 ▸ *e*) (Walshaw & Woolfson, 2003 ▸).

The helix-breaking motif, formed by residues Cys125, Asp126, His127 and Ser128 (Fig. 3 ▸ *b*, inset), causes a rotational displacement of the chain by approximately 90° such that the C-terminal helix of one chain sits above the N-terminal helix of an adjacent chain in the tetramer. Surprisingly, the N-terminal and C-terminal helices are parallel, in contrast to the coiled-coil trimers/tetramers observed for paramyxovirus (Abdella *et al.*, 2020 ▸; Blocquel *et al.*, 2014 ▸; Bloyet *et al.*, 2019 ▸; Bruhn *et al.*, 2014 ▸; Communie *et al.*, 2013 ▸; Cox *et al.*, 2013 ▸; Tarbouriech *et al.*, 2000 ▸) and filovirus (Bruhn *et al.*, 2017 ▸; Yuan *et al.*, 2022 ▸; Zinzula *et al.*, 2019 ▸) phosphoproteins, which twist around each other, placing the N-and C-termini of each chain above those of the adjacent chain. Deletion of a similar motif in the phosphoprotein from measles virus greatly reduced the activity of the virus (Bloyet *et al.*, 2019 ▸).

### 3.3. Analysis of the flexible regions of the phosphoprotein

**Figure 4:**
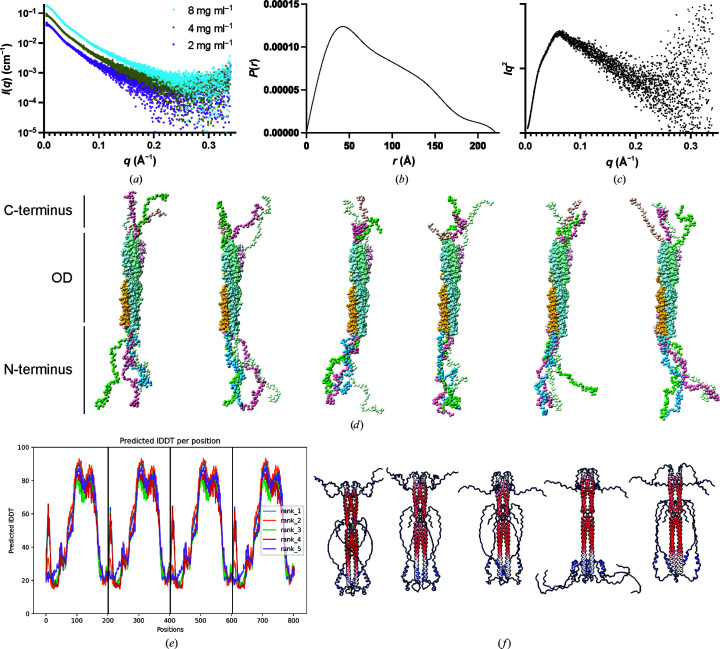
Flexibility analysis of the BoDV-1 phosphoprotein. (*a*) Scattering profile, (*b*) pairwise distribution function and (*c*) Kratky plot of BoDV-1. The *P*(*r*) and Kratky plots are for a protein concentration of 8 mg ml^−1^. (*d*) Models showing possible positions of the N- and C-termini generated with *CORAL*. (*e*, *f*) *AlphaFold*2-predicted IDDT per position and models coloured according to the predicted alignment error from low error (red) to high error (blue).

To understand the flexibility and movement of the regions of the phosphoprotein outside the oligomerization domain, we collected SAXS data at three protein concentrations. The scattering curves for the three concentrations were highly similar; the highest concentration was therefore used as this had the highest signal to noise (Fig. 4 ▸ *a*). A maximum dimension of 221 Å and a radius of gyration of 66 Å were determined for the full-length protein. Analysis of the pairwise distance distribution function shows a distribution with many shorter distances and a gradual fall-off at longer distances; this is indicative of a highly elongated particle (Fig. 4 ▸ *b*). Examination of the Kratky plot (Fig. 4 ▸ *c*) shows a profile characteristic of a particle which is partially unfolded.

To further understand possible locations of the unobserved regions of the phosphoprotein, six models were generated to model the possible positions of each termini. *CORAL* (Petoukhov *et al.*, 2012 ▸) was used with the oligomerization domain as a fixed core and the termini modelled without symmetry (Fig. 4 ▸ *d*). No consensus position of the termini was observed; however, they did tend to be extended in a conformation away from the oligomerization domain.

To test whether *in silico* model prediction suggested a consensus position of the termini, five models were generated with *AlphaFold*2. The predicted accuracy of the models was of high confidence over the oligomerization domain of the phosphoprotein, while both termini were predicted with poor accuracy (Fig. 4 ▸ *e*). Comparison of the structural models show a propensity of residues 40–55 to form helical arrangements that are similar to the phosphoprotein cap observed in the Sendai virus and Nipah virus phosphoprotein structures, although less well ordered (Fig. 4 ▸ *f*; Bruhn *et al.*, 2014 ▸; Tarbouriech *et al.*, 2000 ▸).

### 3.4. Comparison of the oligomerization domain with members of the *Mononegavirales*

**Figure 5:**
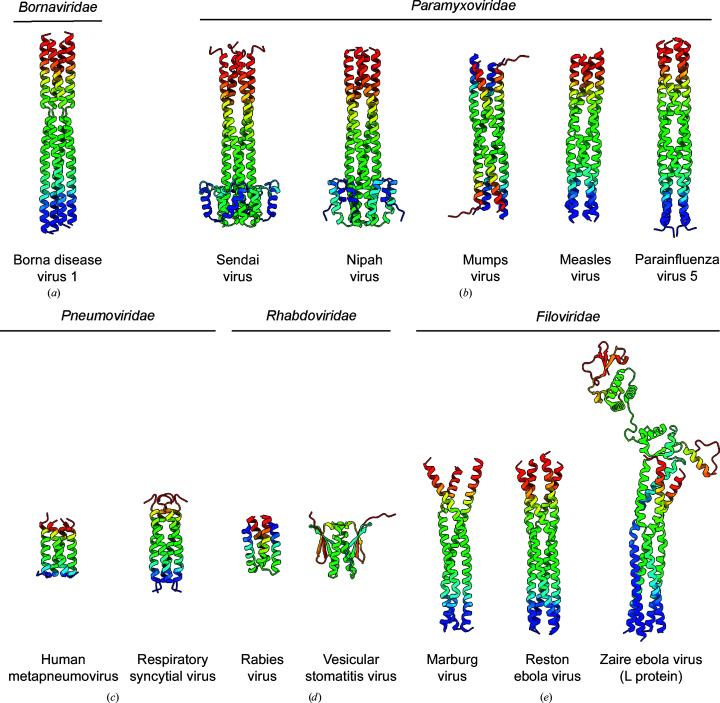
Structural comparison of *Mononegavirales* phosphoproteins. (*a*) The *Bornaviridae* BoDV-1 structure determined in this publication (PDB entry 8bs7). (*b*) *Paramyxoviridae* phosphoprotein structures from Sendai virus (PDB entry 1ezj), Nipah virus (PDB entry 6eb9), mumps virus (PDB entry 4eij), measles virus (PDB entry 3zdo) and parainfluenza virus 5 (PDB entry 6v85). (*c*) *Pneumoviridae* phosphoprotein structures from human metapneumovirus (PDB entry 5oiy) and respiratory syncytial virus (PDB entry 6yp5). (*d*) *Rhabdoviridae* phosphoprotein structures from rabies virus (PDB entry 3i32) and vesicular stomatitis virus (PDB entry 2fqm). (*e*) *Filoviridae* phosphoprotein structures from Marburg virus (PDB entry 5toi), Reston ebola virus (PDB entry 6gbr) and of Zaire ebola virus phosphoprotein in its conformation when bound to the large protein (PDB entry 7yer).

Many structures of the oligomerization domains of phosphoproteins have been determined either alone by X-ray crystallography or in complex with the viral polymerase by cryoelectron microscopy. The BoDV-1 structure determined here is assembled from approximately straight helices, similar to the Sendai and Nipah paramyxovirus structures (Figs. 5 ▸ *a* and 5 ▸ *b*) although lacking the N-terminal capping helices. The tetramer is less twisted than the oligomerization domains of either the measles virus or parainfluenza 5 virus. It is also dissimilar to the antiparallel mumps virus phosphoprotein tetramer.

In contrast to the long helices observed in some species, *Pneumoviridae* phosphoproteins contain much shorter parallel helical oligomerization domains which form tetramers (Fig. 5 ▸ *c*). *Rhabdoviridae* phosphoproteins contain oligomerization domains of a similar length but with unique assemblies. The rabies virus phosphoprotein forms a short helix–turn–helix dimer, while the vesicular stomatitis virus structure forms a dimeric assembly maintained by both α-helices and β-strands (Fig. 5 ▸ *d*).

Filovirus phosphoproteins contain long oligomerization domains formed from helices (Fig. 5 ▸ *e*). Structures of Marburg and Ebola virus phosphoproteins have both been solved by X-ray crystallography and have been shown to form trimers. Recent cryoelectron microscopy data on the Ebola virus L–P complex shows the phosphoprotein to be a tetramer, suggesting there is some plasticity to the oligomeric state or that the trimers were perhaps crystallographic artefacts (Yuan *et al.*, 2022 ▸; Zinzula *et al.*, 2019 ▸).

### 3.5. Conservation of the helix-breaking motif across the *Bornaviridae*

**Figure 6:**
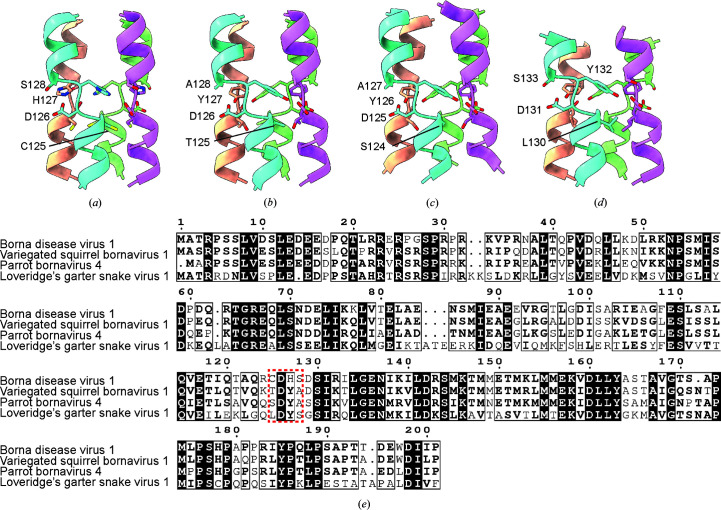
*AlphaFold*2 models of *Bornaviridae* phosphoproteins. *AlphaFold*2 models of (*a*) BoDV-1 (UniProt P0C799), (*b*) variegated squirrel bornavirus 1 (UniProt A0A0H5BWK0), (*c*) parrot bornavirus 4 (UniProt F6JSK3), and (*d*) Loveridege’s garter snake virus 1 (UniProt A0A077ETD9). Chains *A*, *B*, *C* and *D* are coloured teal, wheat, purple and green, respectively. Residues near the helix-breaking motif are shown as sticks. (*e*) A sequence alignment of the four viruses is shown. Residues at the helix-breaking motif are highlighted in the dashed red box.

To investigate the biological conservation of the helix-breaking motif in our model, we generated *AlphaFold*2 predictions for a representative set of other *Bornaviridae* (Figs. 6 ▸ *a*–6 ▸ *d*). We first compared our experimentally determined BoDV-1 structure with the *AlphaFold*2 prediction and an essentially identical motif was observed in both (Figs. 3 ▸ *b* and 6 ▸ *a*). We observe a similar helix-breaking motif in the three structures tested; however, only the aspartic acid at the centre of the motif appears to be strictly conserved (Figs. 6 ▸ *b*–6 ▸ *d*). Residues immediately upstream or downstream of this aspartate appear to have some weak physiochemical conservation.

## 4. Conclusion

The phosphoprotein of *Mononegavirales* plays an essential role in the life cycle of the virus. In this work, we present the structure of the BoDV-1 phosphoprotein, showing it to be a stable tetramer both in solution and when crystallized. Tetramerization is important for at least two reasons. Firstly, during replication a tetrameric phosphoprotein will be able to maintain a higher local concentration of N^0^, which can readily bind RNA that is exposed as part of the replication process, minimizing the chance of recognition by the host. Secondly, the phosphoprotein needs to bind to L as well as to the N-RNA nucleocapsid (via N), so the tetrameric nature of P, containing multiple similar or identical interaction sites, allows it to act as a molecular chaperone binding simultaneously to these different components of the replicase complex. While this work was in preparation, Tarbouriech and coworkers published a similar structure of the BoDV-1 phosphoprotein oligomerization domain complemented by functional studies showing the importance of the helix-breaking motif (Tarbouriech *et al.*, 2022 ▸).

## Supplementary Material

PDB reference: multimerization domain of Borna disease virus 1, 8bs7

## Figures and Tables

**Table 1:** Crystallization

Method	Vapour diffusion
Plate type	SWISSCI 3-drop
Temperature (K)	293
Protein concentration (mg ml^−1^)	6
Buffer composition of protein solution	20 m*M* HEPES pH 7.5, 150 m*M* NaCl, 5%(*v*/*v*) glycerol
Composition of reservoir solution	0.2 *M* ammonium tartrate, 20%(*w*/*v*) PEG 3350
Volume and ratio of drop	200 nl (1:1)
Volume of reservoir (µl)	30

**Table 2:** Data collection and processing Values in parentheses are for the outer shell.

Diffraction source	I03, Diamond Light Source
Wavelength (Å)	0.9763
Temperature (K)	100
Space group	*P*1
*a*, *b*, *c* (Å)	34.87, 35.15, 153.13
α, β, γ (°)	89.97, 90.01, 90.54
Resolution range (Å)	51.0–3.2 (3.31–3.20)
Total No. of reflections	43023 (4156)
No. of unique reflections	11877 (905)
Completeness (%)	93.2 (75.7)
Multiplicity	3.6 (3.5)
〈*I*/σ(*I*)〉	9.9 (2.7)
Overall *B* factor from Wilson plot (Å^2^)	40.9

**Table 3:** Structure solution and refinement Values in parentheses are for the outer shell.

Resolution range (Å)	51.04–3.20 (3.31–3.20)
Completeness (%)	93.3
No. of reflections, working set	11170 (897)
No. of reflections, test set	589 (54)
Final *R*_cryst_	0.33 (0.37)
Final *R*_free_	0.38 (0.45)
No. of non-H atoms	
Protein	4525
R.m.s. deviations	
Bonds (Å)	0.003
Angles (°)	0.46
Average *B* factors (Å^2^)	
Protein	42.8
Ramachandran plot	
Most favoured (%)	99.5
Allowed (%)	0.5
